# Dr. Roget in Reply to Mr. Hume

**Published:** 1812-09

**Authors:** P. M. Roget

**Affiliations:** Bernard-street, Russell-square


### *To the Editors of the Medical and Physical Journal.*

GENTLEMEN,

IN the last Number of your Journal, Mr. Hume complains that, in recommending a test proposed by Dr. Marcet for the detection of minute quantities of arsenic,* I have not paid sufficient attention, or noticed with sufficient accuracy, the method which he had himself pointed out, a few years ago, for a similar purpose. It must appear singular that any imputation of plagiarism should be made against a statement in which the pretended sufferer is not only named, but in which his claims are specifically pointed out, and the document upon which they rest is distinctly referred to. Mr. Hume's words, in the letter I have quoted, are as follows: "Let one grain of white oxide of arsenic, and the same quantity of carbonate of soda be dissolved by boiling, in 10 or 12 ounces of distilled water, which ought to be done in a glass vessel; to this let a small quantity of the nitrate of

* See "A Case of Recovery from the Effects of Arsenic," in the Medico-Chirurgical Transactions, vol. ii. p. 156.

 silver silver be added, and a bright *yellow* precipitate will instantly appear. This is a more decisive test than sulphate of copper, which forms Scheele's green (arseniate of copper); and, though my process answers very well with potass, or even lime-water, yet I am inclined to prefer the common subcarbonate of soda."* His subsequent letters, published in your Journal,† add nothing to this account, except more minute directions respecting the manipulation of the process, particularly the lunar caustic in a dry form. It would have been quite superfluous to have quoted every one of these letters individually, when the whole detail of these operations is contained at length in Dr. Henry's Elements of Experimental Chemistry, to which I took care particularly to refer the reader. The account which I gave of the process was as follows: "Mr. Hume has proposed boiling the suspected matter with a solution of carbonate of potash, and bringing into contact with it a stick of dry nitrate of silver." I certainly was not aware that this passage could be construed into a *misrepresentation*, or *perversion*, or *garbling*, of that of Mr. Hume. That it deserves this censure, however, he infers from the expression "boiling the suspected matter," not occurring in his letter: and it is perfectly true that he does not specify, that, in order to detect the arsenic, we should operate upon the matter *suspected* to contain it.

I confess that, in common with many others, I have misconceived his meaning, if his proposal goes only to the discovery of the presence of that metal in substances where we know already that it exists. The following is the method proposed by Dr. Marcet, as stated in my paper: "Let the fluid, suspected to contain arsenic, be filtered; let the end of a glass rod, wetted with a solution of pure ammonia, be brought into contact with this fluid; and let a clean rod, similarly wetted with a solution of nitrate of silver, be brought into contact with the mixture. If the minutest quantity of arsenic be present, a precipitate of a bright yellow color, inclining to orange, will appear at the point of contact, and will readily subside at the bottom of the vessel." Mr. Hume is not satisfied with my having noticed his process, and acknowledged that it was analogous to Dr. Marcet's, but claims the merit of having suggested the employment of ammonia as answering the same purpose as the soda. I have *carefully* read over the whole of his three letters in your Journal, and cannot discover even a hint that ammonia

* Philosophical Magazine, vol. xxxiii. p. 402.

† Vol. xxiii. p. 448; and xxiv. p. 145 and 403.

might be substituted for soda, not even as tacitly implied in any such general expression as *the other alkalies.* That the employment of ammonia is much more appropriate than any of the alkalies or earths which Mr. Hume has pointed out, is, I think, pretty evident, from its not requiring previous boiling with the suspected matter, and still more from its occasioning no precipitate with nitrate of silver alone, as the fixed alkalies do; a circumstance which, in unskilful hands, would be exceedingly apt to lead to equivocal results.

From his criticisms upon what I have said of our having applied the test to Fowler's solution, in which he observes that the addition of ammonia was unnecessary, it is evident that Mr. Hume has totally misunderstood the object of that experiment, which was tried with a view to ascertain the degree of evidence this compound test would afford of the presence of arsenic, in whatever state of combination that metal might exist. The result of the experiments proved, that even when the ammonia does not contribute to the effect, it does not interfere with it; and that the delicacy of the compound test might therefore be relied upon, when it was applied to a fluid suspected to contain any of the preparations of arsenic.

When the test in question occurred to Dr. Marcet, and when we ascertained together all the collateral circumstances mentioned in my paper respecting the limits of its power, and the agencies of other metallic bodies on the same test, we had not the smallest knowledge or recollection of Mr. Hume's ever having turned his attention to the subject of arsenic; nor did any of the chemical friends, to whom our experiments were shewn, appear to have noticed what he had written on the subject. It was only at the moment of sending our remarks to the press, that Dr. Marcet met with the quotation I have mentioned in Dr. Henry's Elements of Chemistry, and pointed it out to me for the purpose of reference. So far, therefore, from wishing to throw a veil over Mr. Hume's labors, I am exceedingly happy to learn that my paper has been the means of making these tests more generally known, and their merits more generally acknowledged.

I am, Gentlemen,

Your obedient Servant,

P. M. ROGET.

*Bernard-street, Russell-square,*
*August* 6, 1812.

